# 3-Bromo­methyl-4-meth­oxy-2-(2-nitro­phen­yl)-9-phenyl­sulfonyl-9*H*-carbazole

**DOI:** 10.1107/S160053681401143X

**Published:** 2014-05-24

**Authors:** S. Gopinath, K. Sethusankar, Velu Saravanan, Arasambattu K. Mohanakrishnan

**Affiliations:** a Department of Physics, RKM Vivekananda College (Autonomous), Chennai 600 004, India; b Department of Organic Chemistry, University of Madras, Guindy Campus, Chennai 600 025, India

## Abstract

In the title compound, C_26_H_19_BrN_2_O_5_S, the carbazole tricycle is essentially planar, with the largest deviation being 0.126 (3) Å for the C atom connected to the nitro­phenyl group. The carbazole moiety is almost orthogonal to the benzene rings of the adjacent phenyl­sulfonyl and nitro­phenyl groups, making dihedral angles of 85.43 (15) and 88.62 (12)°, respectively. The mol­ecular conformation is stabilized by two C—H⋯O hydrogen bonds involving the sulfone group, which form similar six-membered rings. In the crystal, mol­ecules symmetrically related by a glide plane are linked in *C*(6) chains parallel to [001] by C—H⋯O hydrogen bonds formed with the participation of the nitro group. The chains are reinforced by additional C—H⋯π inter­actions.

## Related literature   

For the uses and biological importance of carbazoles, see: Itoigawa *et al.* (2000 ▶); Ramsewak *et al.* (1999 ▶). For electronic properties and applications, see: Friend *et al.* (1999 ▶); Zhang *et al.* (2004 ▶). For related structures, see: Narayanan *et al.* (2014 ▶); Gopinath *et al.* (2014 ▶). For the Thorpe–Ingold effect, see: Bassindale (1984 ▶). For bond-length distortions, see: Allen *et al.* (1987 ▶). For graph-set notation, see: Bernstein *et al.* (1995 ▶).
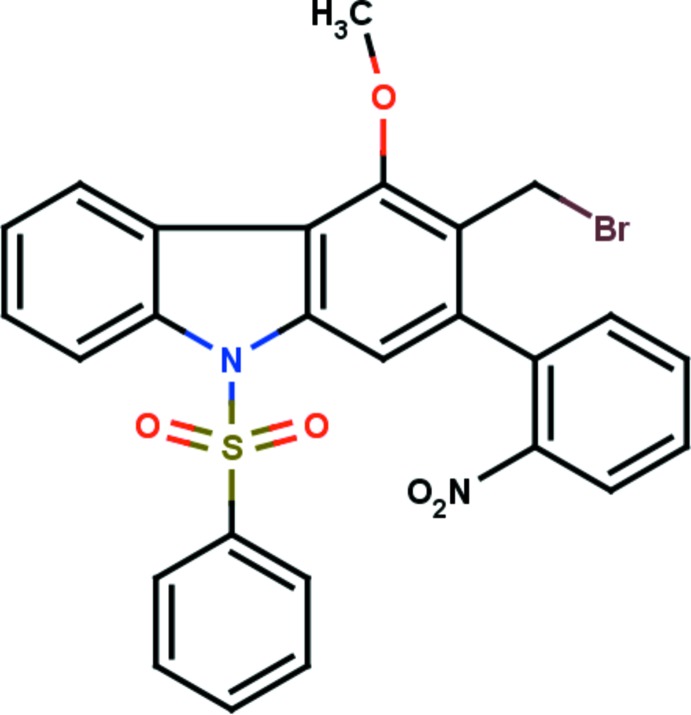


## Experimental   

### 

#### Crystal data  

C_26_H_19_BrN_2_O_5_S
*M* *_r_* = 551.40
Monoclinic, 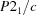

*a* = 10.3176 (4) Å
*b* = 14.4431 (6) Å
*c* = 15.4901 (6) Å
β = 92.329 (2)°
*V* = 2306.40 (16) Å^3^
*Z* = 4
Mo *K*α radiation
μ = 1.92 mm^−1^
*T* = 296 K
0.35 × 0.30 × 0.25 mm

#### Data collection  

Bruker Kappa APEXII CCD diffractometer
Absorption correction: multi-scan (*SADABS*; Bruker, 2008 ▶) *T*_min_ = 0.901, *T*_max_ = 0.905
28756 measured reflections
6652 independent reflections
3939 reflections with *I* > 2σ(*I*)
*R*_int_ = 0.037

#### Refinement  

*R*[*F* ^2^ > 2σ(*F* ^2^)] = 0.050
*wR*(*F* ^2^) = 0.161
*S* = 1.03
6652 reflections
317 parameters
H-atom parameters constrained
Δρ_max_ = 0.42 e Å^−3^
Δρ_min_ = −0.74 e Å^−3^

### 

Data collection: *APEX2* (Bruker, 2008 ▶); cell refinement: *SAINT* (Bruker, 2008 ▶); data reduction: *SAINT*; program(s) used to solve structure: *SHELXS97* (Sheldrick, 2008 ▶); program(s) used to refine structure: *SHELXL97* (Sheldrick, 2008 ▶); molecular graphics: *ORTEP-3 for Windows* (Farrugia, 2012 ▶) and *Mercury* (Macrae *et al.*, 2008 ▶); software used to prepare material for publication: *SHELXL97* and *PLATON* (Spek, 2009 ▶).

## Supplementary Material

Crystal structure: contains datablock(s) global, I. DOI: 10.1107/S160053681401143X/ld2127sup1.cif

Structure factors: contains datablock(s) I. DOI: 10.1107/S160053681401143X/ld2127Isup2.hkl

Supporting information file. DOI: 10.1107/S160053681401143X/ld2127Isup3.cml

CCDC reference: 1003645

Additional supporting information:  crystallographic information; 3D view; checkCIF report

## Figures and Tables

**Table 1:** Hydrogen-bond geometry (Å, °) *Cg*1 is the centroid of the C7–C12 ring.

*D*—H⋯*A*	*D*—H	H⋯*A*	*D*⋯*A*	*D*—H⋯*A*
C2—H2⋯O3	0.93	2.34	2.941 (4)	122
C9—H9⋯O4	0.93	2.32	2.925 (4)	122
C23—H23⋯O1^i^	0.93	2.53	3.264 (4)	136
C22—H22⋯*Cg*1^ii^	0.93	2.95	3.810 (4)	155

Symmetry codes: (i) 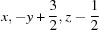; (ii) 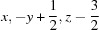.

**Fig. 1.**
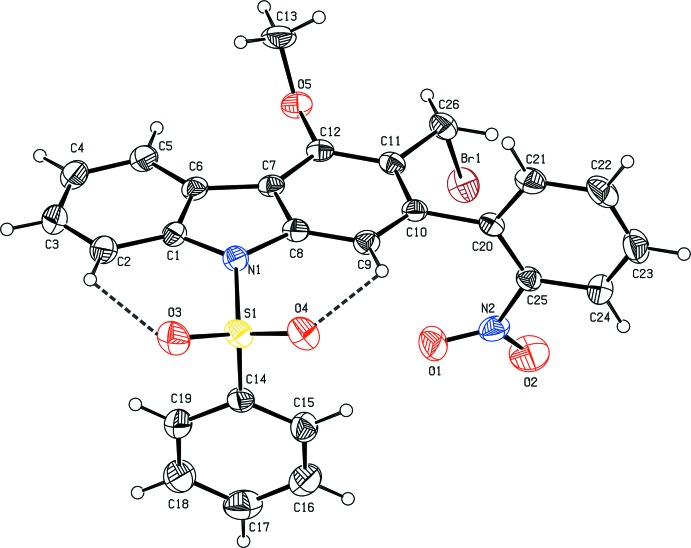
The molecular Structure of the title compound with the atom numbering scheme, displacement ellipsoids are drawn at 30% probability level. H atoms are present as small spheres of arbitrary radius.

**Fig. 2.**
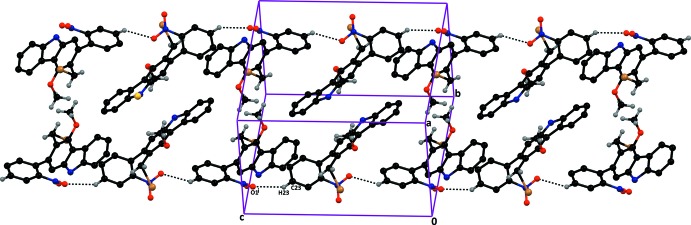
The packing arrangement of the title compound viewed down a axis. The dashed line indicate the C—H···O intermolecular interaction. Symmetry Code: (i) x, -y + 3/2, +z - 1/2

**Fig. 3.**
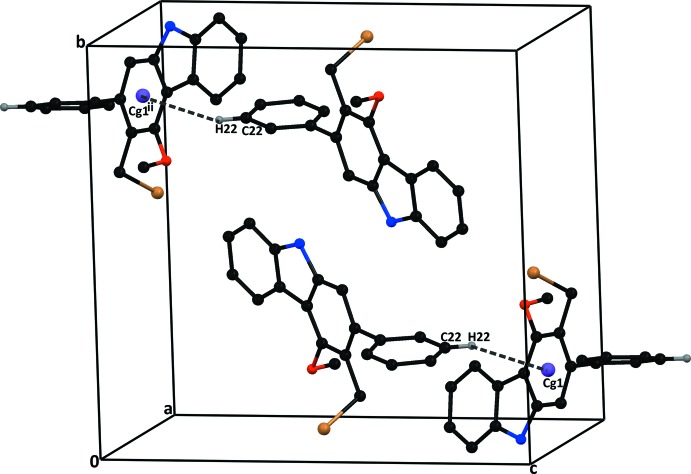
Part of the crystal packing of the title compound viewed down b axis. the dashed lines indicate C22—H22···Cg1ii interactions, where the Cg1 is the centre of the gravity of (C7–C12). Symmetry code: (ii) x, -y + 1/2, +z - 3/2

## supplementary crystallographic information

### 1. Comment

Carbazole and its derivatives have become quite attractive compounds owing to their applications in pharmacy and molecular electronics. It has been reported that carbazole derivatives exihibit various biological activities such as antitumor and antioxidative (Itoigawa, *et al.* 2000), and anti- inflammatory and anti-mutagenic (Ramsewak, *et al.* 1999). They also exhibit electroactivity and luminenscence and are considered to be potential candidates for electronic applications such as colour displays, organic, semi- conductors, laser and solar cells (Friend, *et al.* 1999; Zhang *et al.* 2004).

The title compound, Fig. 1, comprises a carbazole ring system which is attached to a phenylsulfonyl group, a nitrophenyl, a methoxy and a bromomethyl group. The carbazole ring system is essentially planar with maximum deviation of -0.126 (3) Å for the carbon atom C10. The oxygen atom O5 significantly deviates from the carbazole ring by 0.1560 (22) Å. The carbazole ring is almost orthagonal to phenyl ring attached to sulfonyl group and nitrophenyl ring with dihedral angles of 85.43 (15)° and 88.62 (12)°, respectively.

As a result of electron-withdrawing character of phenysulfonyl group, the bond lengths N1–C1 = 1.424 (4) Å and N1–C8 = 1.430 (3) Å in the molecule are longer than the mean value of 1.355 (14) Å (Allen, *et al.* 1987). The atom S1 has a distorted tetrahedral configuration. The widening of angle O3—S1—O4 [120.22 (15)°] and narrowing angle N1—S1—C14 [104.44 (13)°] from the ideal tetrahedral value are attributed to the Thorpe-Ingold effect (Bassindale, *et al.* 1984).

The sum of the bond angles around N1 [353.6°] indicate the *sp*^2^ hybridization. The nitrogen atom N2 is almost in the plane of phenyl ring with the torsional angle of C21–C20–C25–N2 = -179.6 (3)°. The bromine atom forms the torsional angle of C10– C11–C26–Br1 = 88.1 (3)°.

The molecular structure is stabilized by C—H···O hydrogen bonds (Table 1 Fig. 1), which generate two S(6) ring motifs. In the crystal packing, molecules are linked *via* C23—H23···O1^i^ intermolecular hydrogen bonding, which generate *C*(6) infinite chains running parallel to the base vector [0 0 1]. The crystal packing is further stabilized by C22—H22···*Cg*1^ii^ intermolecular interaction, where the *Cg*1 is the centre of gravity of the benzene ring(C7–C12) (Bernstein, *et al.* 1995). The packing view of the title compound shown in the Fig-2 and Fig-3. The symmetry codes are: (i) *x*, -*y* + 1/2 + 1, +*z* - 1/2 (ii) *x*, 3/2 - *y*, -1/2 + *z*

### 2. Experimental

A mixture of 4-methoxy-3-methyl-2-(2-nitrophenyl)-9- (phenylsulfonyl)-9*H*-carbazole(1.65 g, 3.5 mmol) and NBS(0.93 g, 5.25 mmol) in dry CCl4(100 ml) containing a catalytic amount of AIBN(50 mg) was refluxed for 1 h. Then, it was cooled to room temperature and the additional equivalent of NBS(0.93 g, 5.25 mmol) and AIBN(50 mg) were added and allowed to reflux for 1 h. Then the reaction mixture was cooled to room temperature and the floated succinimide was filtered off through Na2So4 pad and washed with hot CCl4 (20 ml). The subsequent removal of solvent *in vacuo* followed by tituration of the crude product with MeOH(10 ml) afforded 3-(bromomethyl)-4-methoxy-2-(2-nitrophenyl)-9-(phenylsulfonyl) -9*H*carbazole(1.71 g, 89%) as a dull white solid. Single crystals suitable for X-ray diffraction was prepared by slow evapouration of a solution of the title compound in methanol at room temperature.

m.p. = 483 K–485 K.

### 3. Refinement

The positions of the hydrogen atoms were localized from the difference electron density maps and the distances were geometrically constrained. The hydrogen atoms bound to the C atoms are treated as riding atoms, with d(C–H) = 0.93 Å and *U*_iso_(H) = 1.2Ueq (*c*) for aromatic and methoxy group, d(C–H) = 0.96 Å and *U*_iso_(H) = 1.5*U*eq(*c*) for the bromomethyl group. The rotation angle for the methyl group was optimized by least squares.

### Crystal data

**Table:**

C_26_H_19_BrN_2_O_5_S	*F*(000) = 1120
*M**_r_* = 551.40	*D*_x_ = 1.588 Mg m^−^^3^
Monoclinic, *P*2_1_/*c*	Mo *K*α radiation, λ = 0.71073 Å
Hall symbol: -P 2ybc	Cell parameters from 6652 reflections
*a* = 10.3176 (4) Å	θ = 2.0–27.0°
*b* = 14.4431 (6) Å	µ = 1.92 mm^−^^1^
*c* = 15.4901 (6) Å	*T* = 296 K
β = 92.329 (2)°	Block, white
*V* = 2306.40 (16) Å^3^	0.35 × 0.30 × 0.25 mm
*Z* = 4	

### Data collection

**Table:**

Bruker Kappa APEXII CCD diffractometer	6652 independent reflections
Radiation source: fine-focus sealed tube	3939 reflections with *I* > 2σ(*I*)
Graphite monochromator	*R*_int_ = 0.037
ω &φ scans	θ_max_ = 30.0°, θ_min_ = 2.0°
Absorption correction: multi-scan (*SADABS*; Bruker, 2008)	*h* = −14→13
*T*_min_ = 0.901, *T*_max_ = 0.905	*k* = −20→19
28756 measured reflections	*l* = −17→21

### Refinement

**Table:**

Refinement on *F*^2^	Primary atom site location: structure-invariant direct methods
Least-squares matrix: full	Secondary atom site location: difference Fourier map
*R*[*F*^2^ > 2σ(*F*^2^)] = 0.050	Hydrogen site location: inferred from neighbouring sites
*wR*(*F*^2^) = 0.161	H-atom parameters constrained
*S* = 1.03	*w* = 1/[σ^2^(*F*_o_^2^) + (0.0816*P*)^2^ + 0.8074*P*] where *P* = (*F*_o_^2^ + 2*F*_c_^2^)/3
6652 reflections	(Δ/σ)_max_ = 0.001
317 parameters	Δρ_max_ = 0.42 e Å^−^^3^
0 restraints	Δρ_min_ = −0.74 e Å^−^^3^

### Special details

**Table:**

Geometry. All e.s.d.'s (except the e.s.d. in the dihedral angle between two l.s. planes) are estimated using the full covariance matrix. The cell e.s.d.'s are taken into account individually in the estimation of e.s.d.'s in distances, angles and torsion angles; correlations between e.s.d.'s in cell parameters are only used when they are defined by crystal symmetry. An approximate (isotropic) treatment of cell e.s.d.'s is used for estimating e.s.d.'s involving l.s. planes.
Refinement. Refinement of *F*^2^ against ALL reflections. The weighted *R*-factor *wR* and goodness of fit *S* are based on *F*^2^, conventional *R*-factors *R* are based on *F*, with *F* set to zero for negative *F*^2^. The threshold expression of *F*^2^ > σ(*F*^2^) is used only for calculating *R*-factors(gt) *etc*. and is not relevant to the choice of reflections for refinement. *R*-factors based on *F*^2^ are statistically about twice as large as those based on *F*, and *R*- factors based on ALL data will be even larger.

### Fractional atomic coordinates and isotropic or equivalent isotropic displacement parameters (Å^2^)

**Table:**

	*x*	*y*	*z*	*U*_iso_*/*U*_eq_	
C1	0.5239 (3)	0.9609 (2)	0.16131 (19)	0.0406 (6)	
C2	0.4792 (3)	1.0177 (2)	0.2262 (2)	0.0545 (8)	
H2	0.4971	1.0808	0.2272	0.065*	
C3	0.4077 (3)	0.9774 (3)	0.2886 (2)	0.0624 (9)	
H3	0.3769	1.0139	0.3328	0.075*	
C4	0.3802 (3)	0.8829 (3)	0.2873 (2)	0.0597 (9)	
H4	0.3326	0.8573	0.3310	0.072*	
C5	0.4221 (3)	0.8274 (2)	0.2225 (2)	0.0488 (7)	
H5	0.4023	0.7645	0.2214	0.059*	
C6	0.4952 (3)	0.8669 (2)	0.15814 (19)	0.0393 (6)	
C7	0.5591 (3)	0.82841 (18)	0.08476 (18)	0.0363 (6)	
C8	0.6265 (3)	0.89955 (18)	0.04518 (18)	0.0368 (6)	
C9	0.7036 (3)	0.88394 (19)	−0.02381 (18)	0.0396 (6)	
H9	0.7454	0.9326	−0.0505	0.048*	
C10	0.7173 (3)	0.79316 (19)	−0.05237 (18)	0.0366 (6)	
C11	0.6474 (3)	0.72050 (18)	−0.01624 (17)	0.0370 (6)	
C12	0.5667 (3)	0.73930 (18)	0.05171 (18)	0.0363 (6)	
C13	0.3650 (3)	0.6679 (3)	0.0589 (2)	0.0654 (10)	
H13A	0.3240	0.7248	0.0748	0.098*	
H13B	0.3216	0.6168	0.0848	0.098*	
H13C	0.3600	0.6614	−0.0028	0.098*	
C14	0.8317 (3)	1.0435 (2)	0.1570 (2)	0.0443 (7)	
C15	0.9401 (3)	1.0058 (2)	0.1228 (2)	0.0546 (8)	
H15	0.9433	0.9957	0.0636	0.065*	
C16	1.0440 (4)	0.9831 (3)	0.1767 (3)	0.0678 (10)	
H16	1.1179	0.9574	0.1541	0.081*	
C17	1.0389 (4)	0.9983 (3)	0.2642 (3)	0.0755 (12)	
H17	1.1099	0.9838	0.3006	0.091*	
C18	0.9288 (4)	1.0349 (4)	0.2979 (3)	0.0779 (12)	
H18	0.9251	1.0441	0.3572	0.093*	
C19	0.8250 (4)	1.0576 (3)	0.2448 (2)	0.0641 (10)	
H19	0.7505	1.0824	0.2675	0.077*	
C20	0.8034 (3)	0.77497 (18)	−0.12588 (17)	0.0372 (6)	
C21	0.7515 (3)	0.7773 (2)	−0.2098 (2)	0.0536 (8)	
H21	0.6637	0.7901	−0.2192	0.064*	
C22	0.8278 (4)	0.7609 (3)	−0.2803 (2)	0.0641 (10)	
H22	0.7906	0.7626	−0.3360	0.077*	
C23	0.9561 (4)	0.7425 (3)	−0.2683 (2)	0.0633 (10)	
H23	1.0071	0.7328	−0.3157	0.076*	
C24	1.0099 (3)	0.7381 (2)	−0.1871 (2)	0.0531 (8)	
H24	1.0975	0.7240	−0.1786	0.064*	
C25	0.9345 (3)	0.75469 (19)	−0.11715 (17)	0.0386 (6)	
C26	0.6569 (3)	0.6230 (2)	−0.0488 (2)	0.0472 (7)	
H26A	0.6094	0.5762	−0.0237	0.057*	
H26B	0.7103	0.6095	−0.0940	0.057*	
N1	0.6006 (2)	0.98366 (15)	0.08997 (16)	0.0407 (5)	
N2	1.0011 (3)	0.7494 (2)	−0.03198 (18)	0.0520 (7)	
O1	0.9650 (3)	0.7991 (2)	0.02487 (15)	0.0771 (8)	
O2	1.0921 (3)	0.6960 (2)	−0.0230 (2)	0.0881 (9)	
O3	0.6318 (3)	1.14928 (15)	0.12702 (17)	0.0634 (6)	
O4	0.7410 (3)	1.08277 (15)	0.00314 (15)	0.0575 (6)	
O5	0.4979 (2)	0.66924 (14)	0.08849 (14)	0.0482 (5)	
S1	0.69953 (8)	1.07490 (5)	0.08859 (5)	0.0460 (2)	
Br1	0.80182 (4)	0.55845 (2)	0.01227 (2)	0.06233 (16)	

### Atomic displacement parameters (Å^2^)

**Table:**

	*U*^11^	*U*^22^	*U*^33^	*U*^12^	*U*^13^	*U*^23^
C1	0.0317 (14)	0.0433 (15)	0.0466 (16)	0.0042 (11)	0.0016 (12)	−0.0051 (13)
C2	0.0420 (18)	0.0529 (19)	0.069 (2)	0.0071 (14)	0.0026 (16)	−0.0164 (17)
C3	0.0446 (19)	0.082 (3)	0.061 (2)	0.0042 (18)	0.0081 (16)	−0.021 (2)
C4	0.0427 (18)	0.085 (3)	0.0519 (19)	−0.0037 (17)	0.0084 (15)	−0.0034 (18)
C5	0.0413 (17)	0.0549 (18)	0.0504 (18)	−0.0038 (14)	0.0027 (14)	−0.0001 (15)
C6	0.0295 (14)	0.0421 (15)	0.0459 (15)	0.0027 (11)	−0.0025 (12)	−0.0004 (12)
C7	0.0283 (13)	0.0367 (14)	0.0434 (15)	0.0008 (10)	−0.0047 (11)	0.0007 (12)
C8	0.0345 (14)	0.0303 (13)	0.0452 (15)	0.0016 (11)	−0.0042 (12)	−0.0011 (11)
C9	0.0397 (15)	0.0339 (14)	0.0453 (16)	−0.0051 (11)	0.0034 (13)	0.0009 (12)
C10	0.0313 (14)	0.0369 (14)	0.0412 (15)	−0.0007 (11)	−0.0038 (11)	−0.0035 (12)
C11	0.0383 (14)	0.0300 (13)	0.0421 (15)	0.0004 (11)	−0.0063 (12)	−0.0038 (11)
C12	0.0344 (14)	0.0313 (13)	0.0427 (15)	−0.0041 (10)	−0.0042 (12)	0.0032 (11)
C13	0.055 (2)	0.077 (2)	0.064 (2)	−0.0356 (19)	0.0000 (18)	−0.0043 (19)
C14	0.0468 (17)	0.0392 (15)	0.0472 (17)	−0.0111 (12)	0.0052 (14)	−0.0028 (13)
C15	0.059 (2)	0.056 (2)	0.0495 (18)	−0.0072 (16)	0.0124 (16)	−0.0026 (15)
C16	0.054 (2)	0.070 (2)	0.080 (3)	0.0002 (18)	0.0105 (19)	0.001 (2)
C17	0.061 (3)	0.088 (3)	0.077 (3)	−0.011 (2)	−0.013 (2)	0.008 (2)
C18	0.068 (3)	0.119 (4)	0.046 (2)	−0.010 (2)	0.0014 (19)	−0.003 (2)
C19	0.052 (2)	0.091 (3)	0.050 (2)	−0.0078 (18)	0.0075 (17)	−0.0119 (18)
C20	0.0411 (15)	0.0327 (13)	0.0376 (14)	−0.0031 (11)	−0.0020 (12)	−0.0027 (11)
C21	0.0506 (18)	0.0586 (19)	0.0505 (18)	−0.0020 (15)	−0.0117 (15)	−0.0032 (15)
C22	0.083 (3)	0.074 (2)	0.0340 (17)	−0.007 (2)	−0.0107 (17)	−0.0052 (16)
C23	0.078 (3)	0.071 (2)	0.0413 (18)	−0.0043 (19)	0.0129 (18)	−0.0095 (16)
C24	0.0504 (19)	0.058 (2)	0.0514 (19)	0.0013 (15)	0.0104 (15)	−0.0059 (15)
C25	0.0405 (15)	0.0413 (15)	0.0337 (14)	−0.0024 (12)	−0.0018 (12)	0.0004 (11)
C26	0.0557 (18)	0.0365 (15)	0.0492 (17)	−0.0043 (13)	0.0000 (14)	−0.0066 (13)
N1	0.0402 (13)	0.0304 (11)	0.0515 (14)	0.0017 (10)	0.0030 (11)	−0.0057 (10)
N2	0.0368 (14)	0.0698 (18)	0.0488 (16)	−0.0042 (13)	−0.0049 (12)	0.0064 (14)
O1	0.0648 (17)	0.127 (3)	0.0389 (13)	0.0121 (16)	−0.0058 (12)	−0.0149 (15)
O2	0.0648 (18)	0.109 (2)	0.088 (2)	0.0272 (17)	−0.0267 (16)	0.0025 (17)
O3	0.0786 (17)	0.0319 (11)	0.0798 (17)	0.0056 (11)	0.0028 (13)	−0.0074 (11)
O4	0.0847 (17)	0.0381 (11)	0.0497 (13)	−0.0083 (11)	0.0030 (12)	0.0064 (9)
O5	0.0499 (12)	0.0393 (11)	0.0554 (12)	−0.0096 (9)	0.0016 (10)	0.0074 (9)
S1	0.0590 (5)	0.0277 (3)	0.0511 (4)	−0.0022 (3)	0.0013 (4)	−0.0011 (3)
Br1	0.0740 (3)	0.0455 (2)	0.0674 (3)	0.01137 (16)	0.00214 (19)	0.00155 (15)

### Geometric parameters (Å, º)

**Table:**

C1—C2	1.390 (4)	C14—S1	1.752 (3)
C1—C6	1.391 (4)	C15—C16	1.371 (5)
C1—N1	1.423 (4)	C15—H15	0.9300
C2—C3	1.369 (5)	C16—C17	1.376 (6)
C2—H2	0.9300	C16—H16	0.9300
C3—C4	1.394 (6)	C17—C18	1.374 (6)
C3—H3	0.9300	C17—H17	0.9300
C4—C5	1.368 (5)	C18—C19	1.365 (6)
C4—H4	0.9300	C18—H18	0.9300
C5—C6	1.397 (4)	C19—H19	0.9300
C5—H5	0.9300	C20—C25	1.385 (4)
C6—C7	1.448 (4)	C20—C21	1.386 (4)
C7—C12	1.389 (4)	C21—C22	1.393 (5)
C7—C8	1.396 (4)	C21—H21	0.9300
C8—C9	1.377 (4)	C22—C23	1.355 (5)
C8—N1	1.429 (4)	C22—H22	0.9300
C9—C10	1.393 (4)	C23—C24	1.355 (5)
C9—H9	0.9300	C23—H23	0.9300
C10—C11	1.402 (4)	C24—C25	1.381 (4)
C10—C20	1.496 (4)	C24—H24	0.9300
C11—C12	1.395 (4)	C25—N2	1.465 (4)
C11—C26	1.500 (4)	C26—Br1	1.971 (3)
C12—O5	1.373 (3)	C26—H26A	0.9300
C13—O5	1.428 (4)	C26—H26B	0.9300
C13—H13A	0.9600	N1—S1	1.668 (2)
C13—H13B	0.9600	N2—O1	1.207 (4)
C13—H13C	0.9600	N2—O2	1.218 (4)
C14—C15	1.370 (5)	O3—S1	1.425 (2)
C14—C19	1.379 (5)	O4—S1	1.412 (2)
			
C2—C1—C6	121.7 (3)	C15—C16—H16	120.0
C2—C1—N1	129.6 (3)	C17—C16—H16	120.0
C6—C1—N1	108.8 (2)	C18—C17—C16	120.1 (4)
C3—C2—C1	117.6 (3)	C18—C17—H17	120.0
C3—C2—H2	121.2	C16—C17—H17	120.0
C1—C2—H2	121.2	C19—C18—C17	120.2 (4)
C2—C3—C4	121.4 (3)	C19—C18—H18	119.9
C2—C3—H3	119.3	C17—C18—H18	119.9
C4—C3—H3	119.3	C18—C19—C14	119.3 (4)
C5—C4—C3	121.0 (3)	C18—C19—H19	120.3
C5—C4—H4	119.5	C14—C19—H19	120.3
C3—C4—H4	119.5	C25—C20—C21	115.8 (3)
C4—C5—C6	118.7 (3)	C25—C20—C10	124.8 (2)
C4—C5—H5	120.7	C21—C20—C10	119.4 (3)
C6—C5—H5	120.7	C20—C21—C22	121.5 (3)
C1—C6—C5	119.6 (3)	C20—C21—H21	119.3
C1—C6—C7	107.4 (2)	C22—C21—H21	119.3
C5—C6—C7	132.9 (3)	C23—C22—C21	120.4 (3)
C12—C7—C8	118.9 (3)	C23—C22—H22	119.8
C12—C7—C6	132.8 (3)	C21—C22—H22	119.8
C8—C7—C6	108.3 (2)	C22—C23—C24	119.8 (3)
C9—C8—C7	122.3 (3)	C22—C23—H23	120.1
C9—C8—N1	129.9 (3)	C24—C23—H23	120.1
C7—C8—N1	107.8 (2)	C23—C24—C25	119.9 (3)
C8—C9—C10	118.0 (3)	C23—C24—H24	120.1
C8—C9—H9	121.0	C25—C24—H24	120.1
C10—C9—H9	121.0	C24—C25—C20	122.6 (3)
C9—C10—C11	121.2 (3)	C24—C25—N2	116.0 (3)
C9—C10—C20	118.6 (2)	C20—C25—N2	121.3 (3)
C11—C10—C20	120.2 (2)	C11—C26—Br1	110.0 (2)
C12—C11—C10	119.2 (2)	C11—C26—H26A	120.0
C12—C11—C26	119.0 (3)	Br1—C26—H26A	81.8
C10—C11—C26	121.8 (3)	C11—C26—H26B	120.0
O5—C12—C7	119.5 (3)	Br1—C26—H26B	78.5
O5—C12—C11	120.3 (2)	H26A—C26—H26B	120.0
C7—C12—C11	120.2 (2)	C1—N1—C8	107.6 (2)
O5—C13—H13A	109.5	C1—N1—S1	123.50 (19)
O5—C13—H13B	109.5	C8—N1—S1	122.6 (2)
H13A—C13—H13B	109.5	O1—N2—O2	123.5 (3)
O5—C13—H13C	109.5	O1—N2—C25	118.6 (3)
H13A—C13—H13C	109.5	O2—N2—C25	117.9 (3)
H13B—C13—H13C	109.5	C12—O5—C13	112.5 (2)
C15—C14—C19	121.0 (3)	O4—S1—O3	120.22 (15)
C15—C14—S1	119.8 (2)	O4—S1—N1	106.54 (14)
C19—C14—S1	119.3 (3)	O3—S1—N1	106.23 (14)
C14—C15—C16	119.3 (3)	O4—S1—C14	109.25 (16)
C14—C15—H15	120.3	O3—S1—C14	108.98 (15)
C16—C15—H15	120.3	N1—S1—C14	104.43 (13)
C15—C16—C17	120.1 (4)		
			
C6—C1—C2—C3	1.5 (5)	C9—C10—C20—C25	90.5 (4)
N1—C1—C2—C3	−179.1 (3)	C11—C10—C20—C25	−92.5 (3)
C1—C2—C3—C4	−0.3 (5)	C9—C10—C20—C21	−90.0 (3)
C2—C3—C4—C5	−1.0 (5)	C11—C10—C20—C21	87.0 (4)
C3—C4—C5—C6	1.0 (5)	C25—C20—C21—C22	−0.5 (5)
C2—C1—C6—C5	−1.5 (4)	C10—C20—C21—C22	179.9 (3)
N1—C1—C6—C5	179.0 (3)	C20—C21—C22—C23	−0.3 (6)
C2—C1—C6—C7	−178.4 (3)	C21—C22—C23—C24	1.3 (6)
N1—C1—C6—C7	2.0 (3)	C22—C23—C24—C25	−1.6 (6)
C4—C5—C6—C1	0.2 (4)	C23—C24—C25—C20	0.7 (5)
C4—C5—C6—C7	176.2 (3)	C23—C24—C25—N2	−179.4 (3)
C1—C6—C7—C12	178.5 (3)	C21—C20—C25—C24	0.3 (4)
C5—C6—C7—C12	2.1 (5)	C10—C20—C25—C24	179.8 (3)
C1—C6—C7—C8	0.6 (3)	C21—C20—C25—N2	−179.5 (3)
C5—C6—C7—C8	−175.8 (3)	C10—C20—C25—N2	0.0 (4)
C12—C7—C8—C9	−2.1 (4)	C12—C11—C26—Br1	−92.0 (3)
C6—C7—C8—C9	176.1 (3)	C10—C11—C26—Br1	88.1 (3)
C12—C7—C8—N1	178.8 (2)	C2—C1—N1—C8	176.7 (3)
C6—C7—C8—N1	−2.9 (3)	C6—C1—N1—C8	−3.9 (3)
C7—C8—C9—C10	−2.2 (4)	C2—C1—N1—S1	24.2 (4)
N1—C8—C9—C10	176.6 (3)	C6—C1—N1—S1	−156.4 (2)
C8—C9—C10—C11	4.5 (4)	C9—C8—N1—C1	−174.8 (3)
C8—C9—C10—C20	−178.5 (2)	C7—C8—N1—C1	4.2 (3)
C9—C10—C11—C12	−2.5 (4)	C9—C8—N1—S1	−22.0 (4)
C20—C10—C11—C12	−179.4 (2)	C7—C8—N1—S1	157.0 (2)
C9—C10—C11—C26	177.4 (3)	C24—C25—N2—O1	147.7 (3)
C20—C10—C11—C26	0.5 (4)	C20—C25—N2—O1	−32.5 (4)
C8—C7—C12—O5	−178.1 (2)	C24—C25—N2—O2	−30.9 (4)
C6—C7—C12—O5	4.2 (5)	C20—C25—N2—O2	148.9 (3)
C8—C7—C12—C11	4.2 (4)	C7—C12—O5—C13	81.0 (3)
C6—C7—C12—C11	−173.5 (3)	C11—C12—O5—C13	−101.3 (3)
C10—C11—C12—O5	−179.6 (2)	C1—N1—S1—O4	−170.5 (2)
C26—C11—C12—O5	0.5 (4)	C8—N1—S1—O4	41.0 (3)
C10—C11—C12—C7	−2.0 (4)	C1—N1—S1—O3	−41.2 (3)
C26—C11—C12—C7	178.1 (3)	C8—N1—S1—O3	170.3 (2)
C19—C14—C15—C16	−0.9 (5)	C1—N1—S1—C14	73.9 (2)
S1—C14—C15—C16	178.7 (3)	C8—N1—S1—C14	−74.6 (2)
C14—C15—C16—C17	−0.1 (6)	C15—C14—S1—O4	−18.4 (3)
C15—C16—C17—C18	1.1 (7)	C19—C14—S1—O4	161.3 (3)
C16—C17—C18—C19	−1.0 (7)	C15—C14—S1—O3	−151.5 (3)
C17—C18—C19—C14	0.0 (7)	C19—C14—S1—O3	28.1 (3)
C15—C14—C19—C18	0.9 (5)	C15—C14—S1—N1	95.3 (3)
S1—C14—C19—C18	−178.7 (3)	C19—C14—S1—N1	−85.1 (3)

### Hydrogen-bond geometry (Å, º)

*Cg*1 is the centroid of the C7–C12 ring.

**Table:**

*D*—H···*A*	*D*—H	H···*A*	*D*···*A*	*D*—H···*A*
C2—H2···O3	0.93	2.34	2.941 (4)	122
C9—H9···O4	0.93	2.32	2.925 (4)	122
C23—H23···O1^i^	0.93	2.53	3.264 (4)	136
C22—H22···*Cg*1^ii^	0.93	2.95	3.810 (4)	155

Symmetry codes: (i) *x*, −*y*+3/2, *z*−1/2; (ii) *x*, −*y*+1/2, *z*−3/2.
